# Expression Profiling of Human Genetic and Protein Interaction Networks in Type 1 Diabetes

**DOI:** 10.1371/journal.pone.0006250

**Published:** 2009-07-17

**Authors:** Regine Bergholdt, Caroline Brorsson, Kasper Lage, Jens Høiriis Nielsen, Søren Brunak, Flemming Pociot

**Affiliations:** 1 Hagedorn Research Institute and Steno Diabetes Center, Gentofte, Denmark; 2 Center for Biological Sequence Analysis, Technical University of Denmark, Lyngby, Denmark; 3 Pediatric Surgical Research Laboratories, Massachusetts General Hospital, Harvard Medical School, Boston, Massachusetts, United States of America; 4 Broad Institute of MIT and Harvard, Seven Cambridge Center, Cambridge, Massachusetts, United States of America; 5 Department of Medical Biochemistry and Genetics, University of Copenhagen, Copenhagen, Denmark; 6 University of Lund/Clinical Research Centre (CRC), Malmø, Sweden; 7 Department of Biomedical Science, University of Copenhagen, Copenhagen, Denmark; Mayo Clinic College of Medicine, United States of America

## Abstract

Proteins contributing to a complex disease are often members of the same functional pathways. Elucidation of such pathways may provide increased knowledge about functional mechanisms underlying disease. By combining genetic interactions in Type 1 Diabetes (T1D) with protein interaction data we have previously identified sets of genes, likely to represent distinct cellular pathways involved in T1D risk. Here we evaluate the candidate genes involved in these putative interaction networks not only at the single gene level, but also in the context of the networks of which they form an integral part. mRNA expression levels for each gene were evaluated and profiling was performed by measuring and comparing constitutive expression in human islets versus cytokine-stimulated expression levels, and for lymphocytes by comparing expression levels among controls and T1D individuals. We identified differential regulation of several genes. In one of the networks four out of nine genes showed significant down regulation in human pancreatic islets after cytokine exposure supporting our prediction that the interaction network as a whole is a risk factor. In addition, we measured the enrichment of T1D associated SNPs in each of the four interaction networks to evaluate evidence of significant association at network level. This method provided additional support, in an independent data set, that two of the interaction networks could be involved in T1D and highlights the following processes as risk factors: oxidative stress, regulation of transcription and apoptosis. To understand biological systems, integration of genetic and functional information is necessary, and the current study has used this approach to improve understanding of T1D and the underlying biological mechanisms.

## Introduction

Currently, genome-wide association studies in complex diseases are producing an unprecedented amount of genetic data. Complex traits like Type 1 Diabetes (T1D) are influenced by multiple genes interacting with each other to confer susceptibility and/or protection. However, identifying the individual components can be difficult because each only contributes weakly to the pathology. Alternatively, identification of entire cellular systems involved in a particular disease could be attempted. Such a strategy should be feasible in many different complex diseases since most genes exert their function as members of molecular machines where groups of proteins contributing to disease can be expected to be members of the same functional pathways [1], [2], [3], [4], [5], [6]. Analysis of an entire disease-related system might provide insight to the molecular etiology of the disease that would not emerge from isolated functional studies of single genes.

We have previously in a large T1D linkage data set demonstrated statistical evidence for gene-gene interactions [7]. The data set comprised data from 1,321 affected sib pairs genotyped for 298 microsatellite markers [7], [8]. By an integrative approach combining genetic data and high-confidence (human) protein interaction networks, we identified four protein interaction networks significantly enriched in proteins from the predicted genetic interactions. This supported interaction in biological pathways. For each of these networks the identified protein or proteins were viewed in a biological context [7].

However, further functional and genetic evaluation is necessary to confirm the involvement of these interactions in T1D, elucidate the biological mechanisms of these networks and to identify the strongest risk factors amongst the network members. If several members of the same network can be shown to be likely risk factors in independent data this would support that the interaction networks as such are risk factors and serve as a validation of the genetic interactions previously identified. In the current study we use independent approaches for evaluating interaction networks and identifying the strongest risk factors amongst network members. We have used available T1D genome-wide association scan data for evaluation of whether entire interaction networks could be significantly associated with T1D. Furthermore, we performed expression profiling of identified genes. The hypothesis behind this is that expression levels may act as intermediate phenotypes between DNA sequence variation and more complex disease phenotypes and that evaluation of the expression of candidate genes in relevant tissue and/or disease models may provide a means for identifying those with a functional implication in T1D pathogenesis.

## Results and Discussion

We have evaluated expression levels of candidate genes previously identified through genetic and protein interaction analyses [7]. The selected candidate genes originate from linkage regions predicted to genetically interact, and are functionally supported by evidence for physical interaction at the level of protein complexes. Four functional interaction networks (A–D) containing 30 proteins presumed to be responsible for the genetic interactions were previously obtained [7], and these four putative pathways and their 30 members were further evaluated in the present study.

In a model of T1D, expression levels were evaluated in human islet preparations, representing the target organ, as well as in human lymphocytes representing the effector cells in T1D. Expression profiling in human islets was performed by comparing the constitutive expression versus cytokine-stimulated expression levels. Gene expression levels in lymphocytes were compared among controls and T1D individuals.

Additional support for individual genes and genetic interactions in the networks comes from evidence for genetic association. The Wellcome Trust Case Control Consortium (WTCCC) has made the results of their large genome-wide association study of T1D and other diseases publicly available (www.wtccc.org) [9]. In this data set we searched for T1D associated SNPs in the 30 candidate genes located in the four interaction networks. To test for combined evidence for T1D association of the protein networks we measured the over-representation (enrichment) of significant SNPs associated with T1D in the four interaction networks, compared to randomly generated networks with similar properties. For each network we tested the enrichment of SNPs in the best 0.1 percentile, 1 percentile and 5 percentile of the WTCCC data for T1D. A nominal P-value and an adjusted P-value was determined for enrichment at each of those thresholds by comparing to 1,000 randomly generated networks with an equal number of proteins and proteins encoded by genes of similar size to the actual test genes.

**Figure 1:**
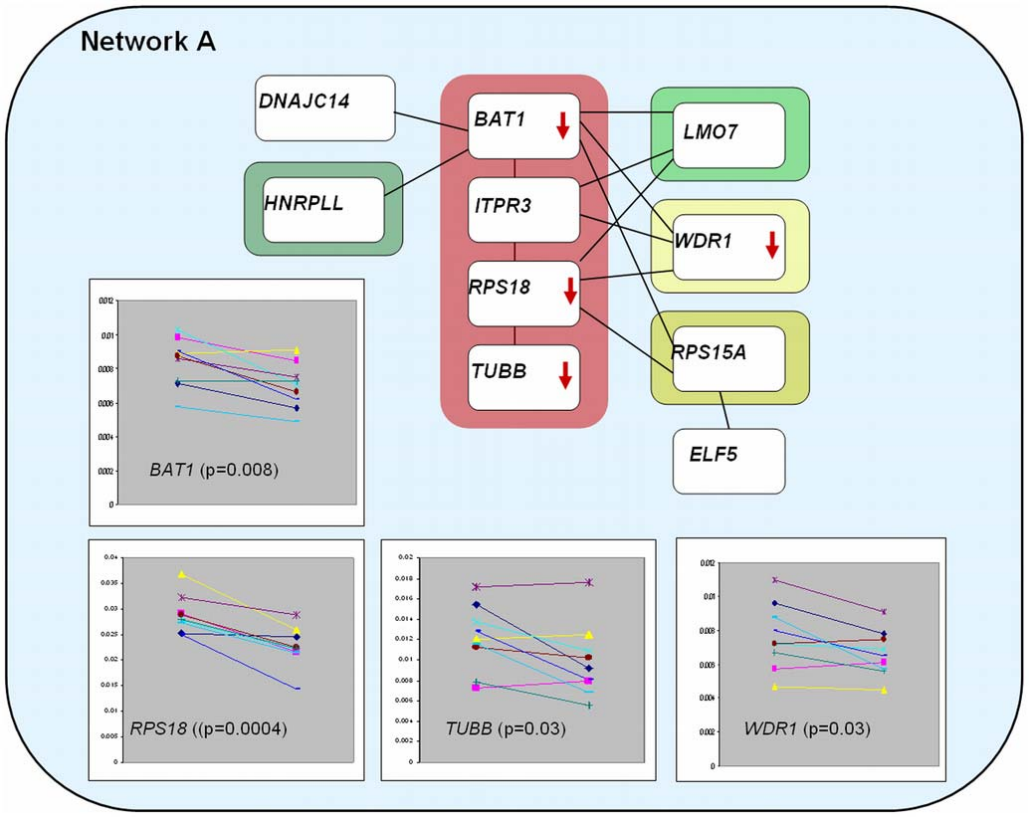
Overview of predicted interactions in network A. Protein-protein interactions in this network originates from predicted genetic interactions between the HLA-region on chromosome 6 (*BAT1, ITPR3, RPS18* and *TUBB*) and chromosomal regions on chromosome 2 (around *D2S177, HNRPL*), chromosome 13 (*D13S170, LMO7*), chromosome 4 (*D4S403, WDR1*) and chromosome 16 (*D16S287, RPS15A*), respectively. The *ELF5* gene is positioned on chromosome 11. Red arrows and corresponding plots refer to four genes that were demonstrated significantly down-regulated in human pancreatic islets upon cytokine-stimulation. In the plots nodes to the left represent expression levels for all nine donors in un-stimulated condition, whereas nodes to the right represent cytokine-stimulated expression levels.

Interaction network A, table 1 and figure 1, represents genetic interactions between the HLA region on chromosome 6 and a region on chromosome 13, a region on chromosome 4, as well as regions on chromosome 16 and 2. Based on validated protein-protein interactions the proteins/genes responsible for these interactions in network A are the four HLA region genes, *BAT1* (Spliceosome RNA helicase, HLA-B associated transcript-I), *ITPR3* (Inositol 1,4,5-triphosphate receptor type 3), *RPS18* (40S ribosomal protein S18) and *TUBB* (Tubulin beta-2 chain) interacting with the *LMO7* (LIM domain only protein 7) gene on chromosome 13, the *WDR1* (WD repeat domain I) gene on chromosome 4, the *RPS15A* (40S ribosomal protein S15a) on chromosome 16 and the *HNRPLL* (Heterogeneous nuclear ribonucleoprotein L-like, stromal RNA-regulating factor) on chromosome 2, as well as two genes directly interacting from other chromosomal regions, *DNAJC14* (Nuclear protein Hcc-1, proliferation associated cytokine-inducible protein CIP29) and *ELF5* (ETS-related transcription factor Elf-5). Network A is significantly enriched, after correction for multiple testing, for SNPs in the 0.1 percentile and the 1 percentile, and borderline significant for SNPs in the best 5 percentile of the WTCCC study, table 2, indicating that the interaction network as a whole is a risk factor in T1D and further supporting the genetic interactions observed in previous work. For genes in network A no significant differences in expression levels between lymphocytes from eight newly diagnosed T1D patients and nine control individuals was identified. For the nine human islet preparations we found that four of the genes demonstrated significant down-regulation upon cytokine-stimulation. These were *BAT1, RPS18* and *TUBB* from the HLA-region and the *WDR1* gene, on the short arm of chromosome 4, a gene involved in actin binding, table 1 and figure 1. This gives further support to these four genes as functionally relevant in this model of T1D (human islets stimulated with cytokines). Neither the *RPS18* nor the *TUBB* gene has known functional roles in relation to T1D. The *BAT1* gene has been designated HLA-B associated transcript and *BAT1* is a negative regulator of inflammation [10], probably affecting production of TNFα, IL-1β and IL-6. WD-repeats are involved in protein-protein interactions and have been shown to be involved in regulation of transcription, mRNA modification and transmembrane signaling, and mutations in the Wdr1 gene has in a mouse model been shown to be related to auto-inflammation [11], [12]. Such effects may be relevant in T1D.

**Table 1:** Expressional profiling of thirty candidate genes divided into four interaction networks (A–D) demonstrating genetic and protein interactions.

Gene symbol	Chr.position	Gene name	P-value
**Network A.**			
*BAT1*	6p21.33	Spliceosome RNA helicase BAT1 HLA-B associated transcript-1	*P = 0.008*
*ITPR3*	6p21.31	Inositol 1,4,5-trisphosphate receptor type 3	*NS*
*RPS18*	6p21.32	40S ribosomal protein S18 Ke-3	*P = 0.0004*
*TUBB*	6p21.33	Tubulin beta-2 chain	*P = 0.03*
*HNRPLL*	2p22.1	Heterogeneous nuclear ribonucleoprotein L-like Stromal RNA-regulating factor	*NS*
*LMO7*	13q22.2	LIM domain only protein 7 LOMP F-box only protein 20	*NS*
*WDR1*	4p16.1	WD repeat domain 1 WDR1, transcript variant 1	*P = 0.03*
*RPS15A*	16p12.3	40S ribosomal protein S15a	*NS*
*DNAJC14*	12q13.2	Nuclear protein Hcc-1 Proliferation associated cytokine-inducible protein CIP29	*NS*
*ELF5*	11p13	ETS-related transcription factor Elf-5 E74-like factor 5	*NS*
**Network B.**			
*RDBP*	6p21.3	RD RNA-binding protein, major histocompatibility complex gene RD	*NS*
*GTF2H4*	6p21.3	Basic transcription factor 2 89 kDa subunit, DNA excision repair protein ERCC-3	*NS*
*RRN3*	16p13.11	RNA polymerase I-specific transcription initiation factor	*NS*
*ERCC4*	16p13.12	DNA excision repair protein, DNA repair endonuclease	*NS*
*TAF1A*	1q41	TATA box binding protein TBP-associated factor, RNA polymerase I	*P = 0.04*
*TYW3*	1p31.1	tRNA-yW synthesizing protein 3 homolog	*NS*
*GUF1*	4p13	GTP-binding protein GUF1 homolog, GTPase of unknown function	*NS*
**Network C.**			
*MOG*	6p22.1	Myelin-oligodendrocyte glycoprotein precursor	*NS*
*APLP2*	11q24.3	Amyloid-like protein 2 precursor APPH	*NS*
*NTRI*	11q25	Neurotrimin precursor hNT	*NS*
**Network D.**			
*DDX52*	17q12	Probable ATP-dependent RNA helicase DDX52 DEAD box protein 52	*NS*
*RPL23A*	17q11.2	60S ribosomal protein L23a	*NS*
*NPM1*	5q35.1	Nucleophosmin NPM Nucleolar phosphoprotein B23	*P = 0.004*
*RPL26L1*	5q35.1	60S ribosomal protein L26-like 1	*NS*
*PRDX1*	1p34.1	Natural killer cell-enhancing factor A, Peroxiredoxin-1	*P = 0.003*
*RPS7*	2p25.3	40S ribosomal protein S7	*NS*
*NGB*	14q24.3	Neuroglobin	*NS*
*FLOT1*	6p21.33	Flotillin 1, integral membrane component of caveolae	*NS*
*SESN1*	6q21	Sestrin-1 p53-regulated protein PA26	*NS*
*SESN2*	1p35.3	Sestrin-2, hypoxia induced gene 95 Hi95	*NS*

Only p-values below 0.05 are considered statistically significant and are included in the table. Non-significant is indicated by NS. *TAF1A* in module B demonstrated differential expression in lymphocytes (T1D vs. controls) whereas the other significant p-values correspond to comparisons of un-stimulated vs. cytokine-stimulated human pancreatic islets.

**Table 2:** The enrichment of the four interaction networks for significant SNPs associated with T1D in the WTCCC study is measured.

	0.1 Percentile	1 Percentile	5 Percentile
**Network A**			
P-value	<0.001	<0.001	0.005
Adjusted P-value	<0.012	<0.012	0.06
**Network B**			
P-value	0.024	0.193	0.648
Adjusted P-value	0.28	1.0	1.0
**Network C**			
P-value	1.0	0.591	0.301
Adjusted P-value	1.0	1.0	1.0
**Network D**			
P-value	0.003	0.121	0.172
Adjusted P-value	0.036	1.0	1.0

P-values refer to a comparison with randomly generated modules with similar properties. P-values and adjusted p-values corrected for multiple testing are provided for all three percentiles of SNPs in each network.

**Figure 2:**
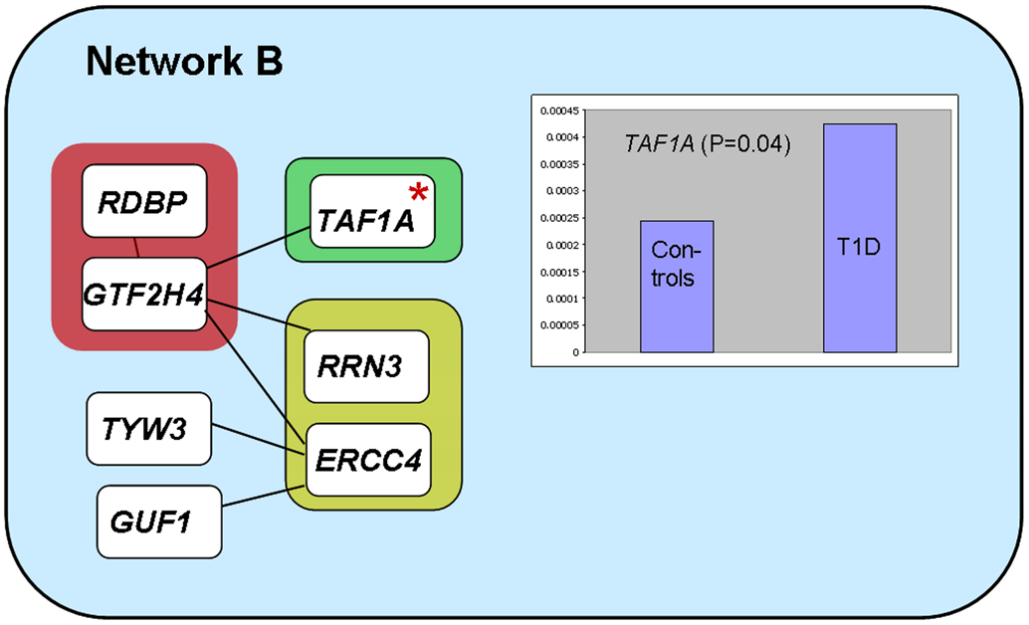
Protein-protein interactions in network B, originating from predicted genetic interactions between the HLA region on chromosome 6 (*RDBP* and *GTF2H4*) and chromosomal regions on chromosome 16 (*D16S287, RRN3* and *ERCC4)*) and chromosome 1 (*D1S229, TAF1A*). The *TAF1A* gene demonstrated significantly higher expression in lymphocytes from T1D patients compared to lymphocytes from control individuals. The *TYW3* and *GUF1* are positioned on chromosome 1p31.1 and 4p13, respectively.

Interaction network B, table 1 and figure 2, consists of two HLA region genes, *RDBP* (RD RNA-binding protein, MHC complex gene RD) and *GTF2H4* (General transcription factor II H) interacting with two genes from a region on chromosome 16, *RRN3* (RNA polymerase I-specific transcription initiation factor) and *ERCC4* (DNA excision repair protein, DNA repair endonuclease) and the *TAF1A* (TATA box binding protein (TBP)-associated factor, RNA polymerase I) gene on chromosome 1. The *ERCC4* gene furthermore directly interacts with the *TYW3* (tRNA–yW synthesizing protein 3 homolog) and the *GUF1* (GTP-binding protein GUF1 homolog, GTPase of unknown function) genes from other regions. Comparing the constitutive expression level of these genes in human pancreatic islets with the level after cytokine-exposure of the islets did not identify significant differences. When comparing expression levels between newly diagnosed T1D patients and controls, we demonstrated a significantly higher expression level of the *TAF1A* gene in the T1D patients, table 1 and figure 2. The *TAF1A* gene on chromosome 1 encodes a transcription factor involved in RNA synthesis. It has not been implicated in T1D before and the effect of different expression levels in relation to disease state in human lymphocytes is not clear, neither is the putative functional effect on the other network B genes by this difference. No enrichment of T1D associated SNPs in this interaction network could be demonstrated, providing no further genetic support to this network.

Interaction network C contains only three genes and originates from an identified gene-gene interaction between the HLA-region and a region on chromosome 11. Genes predicted to be responsible for this interaction is the *MOG* (Myelin-oligodendrocyte glycoprotein precursor) gene on chromosome 6 and the *APLP2* (Amyloid-like protein 2 precursor (APPH)) and *NTRI* (Neurotrimin precursor (hNT)) genes on chromosome 11, table 1. No significant differences in either of the tissues/model systems were demonstrated for these genes, not providing any functional support from expression studies for the network C genes, also no enrichment of T1D associated SNPs was observed in this network.

**Figure 3:**
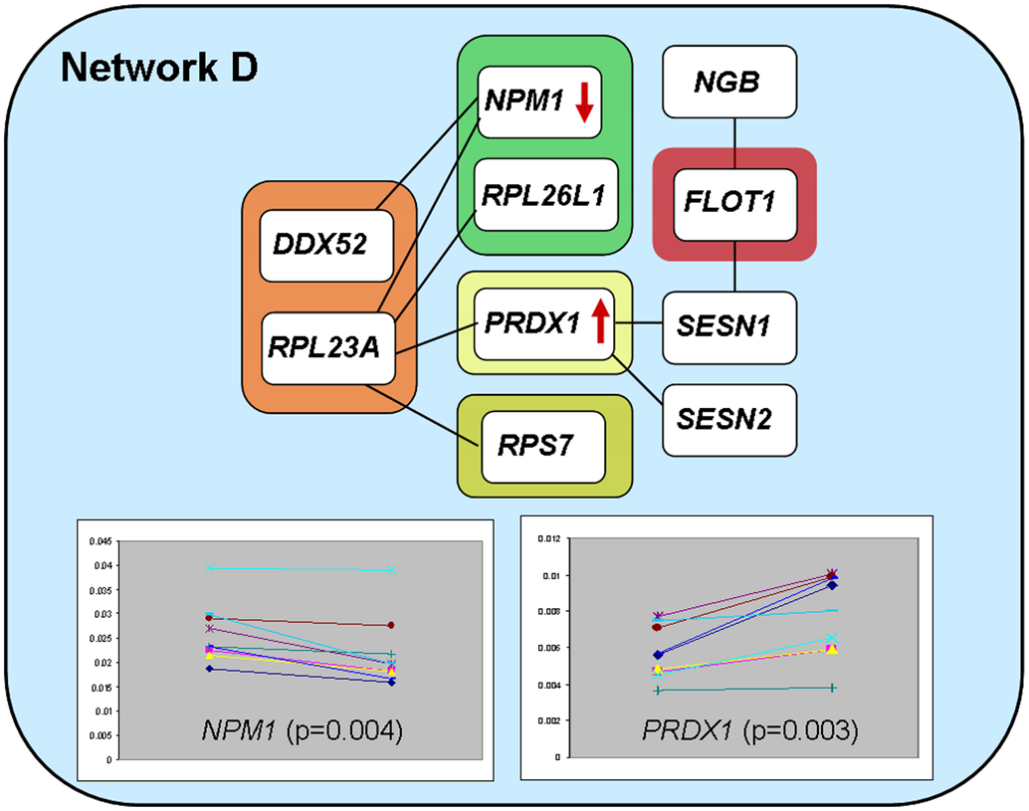
Overview of predicted interactions in network D. Protein-protein interactions originates from identified genetic interactions between a region on chromosome 17 (around *D17S798, DDX52* and *RPL23A*) and regions on chromosome 5 (*D5S429, NPM1* and *RPL26L1*), chromosome 1 (*D1S197, PRDX1*) and chromosome 2 (*D2P25, RPS7*), respectively. Through the *PRDX1* gene in the *D1S197* region the network is linked to the *FLOT1* gene in the HLA-region. The *NGB, SESN1* and *SESN2* genes are positioned on chromosomes 14q24.3, 6q21 and 1p35.3, respectively. No differential expression in lymphocytes were identified, but the *NPM1* gene was significantly down-regulated and the *PRDX1* gene significantly up-regulated by cytokine-stimulation of human pancreatic islets.

Interaction network D, table 1 and figure 3, originates from predicted genetic interactions among a region on chromosome 17, and markers on chromosomes 5, 1 and 2. Candidate genes presumed to be responsible for the interactions are *DDX52* (Probable ATP-dependent RNA helicase DDX52, DEAD box protein 52) and *RPL23A* (60S ribosomal protein L23a) on chromosome 17, *NPM1* (Nucleophosmin, nucleolar phosphoprotein B23) and *RPL26L1* (60S ribosomal protein L26-like I) on chromosome 5, *PRDX1* (Natural killer cell-enhancing factor A, Peroxiredoxin-I) on chromosome 1 and *RPS7* (40S ribosomal protein S7) on chromosome 2. The *PRDX1* gene additionally interacts with the *SESN2* (Sestrin 2, hypoxia induced gene 95) and *SESN1* (Sestrin 1, p53-regulated protein PA26) genes, of which the latter further interacts with the *FLOT1* (Flotillin I, integral membrane component of caveolae) gene from the HLA region. Interaction network D is significantly enriched for SNPs in the best 0.1 percentile, table 2, indicating that genetic variations in this network could also contribute to T1D susceptibility. No differences in the lymphocyte expression studies were demonstrated. Comparing human islets expression profiles, we demonstrated that *NPM1*, a gene involved in ribosomal protein assembly and transport was significantly down-regulated upon stimulation of islets with a mixture of cytokines and the *PRDX1* gene, encoding a natural killer cell enhancing factor, to be significantly up-regulated after such stimulation, when compared to constitutive expression, table 1 and figure 3. Natural killer cell enhancing factors have been described as important for different cells in their defense against oxidants/oxidative stress [13], [14]. An up-regulation of the *PRDX1* encoded protein could be part of a defense mechanism in the beta cell against oxidative stress. The *NPM1* gene has been shown to be important in certain cancer forms, and it has been described that inhibition of the expression of this gene may cause other proteins, e.g. STAT5A to act as a tumor suppressor [15]. This network, build on genetic interactions between chromosome 17 and chromosomes 5, 1 and 2, may be interesting in individuals with a low HLA risk, even though a second order interaction with a gene from the HLA region was found in the protein interactions. The network could point at underlying mechanisms and putatively important combinations of genes responsible for T1D in such individuals. A stronger effect of non-HLA genes could be expected in individuals carrying a lower HLA risk, as indicated by a recent T1D genome-wide association scan and meta-analysis [16].

In three out of four interaction networks our attempts to functionally characterize the candidate genes by expressional profiling have identified novel genes for further analysis. The third interaction network, network C, did not reveal any differentially expressed genes. Genetic support by evaluation of whether networks were enriched in T1D associated SNPs was obtained for network A and to a lesser degree for network D, highlighting these two interaction networks as the most important.

A recently published study evaluated changes at the proteome level after cytokine stimulation of INS-1E cells (a rat tumor beta-cell line serving as an in vitro model for T1D) [17]. Among the proteins that changed expression levels after 4 hours of stimulation with IL-1β and IFN-γ were the rat gene products of the *WDR1* and *NPM1* genes and after 24 hours the *PRDX1* protein was highly up-regulated. In that study a large protein interaction network containing many of the differentially expressed proteins including *WDR1, NPM1* and *PRDX1* was identified [17]. Despite use of different species and model systems and unknown dynamic differences in the transcriptome and proteome we find it of interest that these three genes were pin-pointed as functionally relevant in the current study as well as in the study by D'Hertog [17]. The likelihood of an overlap between three genes in these two studies has a P-value of<0.05, as calculated using hypergeometric statistics.

To understand biological systems integration of genetic and functional information is necessary. This includes studies of gene-gene and protein-protein interactions and transcriptional or proteome profiling. In a previous study we identified genetic interactions observed in T1D and explained their functionality by using an approach for integrating protein-protein interactions generating protein interaction networks. In this work we validated the discovered networks and analyzed their functionality by expressional profiling in relevant target tissue and by using SNP association data. Protein interaction data generally are noisy and databases probably contain many false positives. The system used in the current study is, however, rigorously quality controlled to only include interactions that have been replicated in independent screens [18].

GO (gene ontology) terms (www.geneontology.org) for molecular function and biological processes of interaction networks A and D and the differentially expressed genes in particular support that oxidative stress and regulation of transcription and apoptosis are of relevance for beta-cell destruction in T1D pathogenesis, and points directly at these pathways as the most important. Despite the overall impression from recent genome-wide association studies [9], [19], [20] that genes of importance in T1D are mainly immune system genes and not beta-cell genes, it seems that by integrative genomics also genes involved in the genetic disposition to e.g. cytokine induced beta-cell death by apoptosis can be identified.

Only few studies have addressed genetic interaction in T1D and focused on interactions between classical disease loci [21], [22], [23], and in the case of high risk HLA class II genotypes and *PTPN22* a less than multiplicative association has been demonstrated in T1D and rheumatoid arthritis [16], [24], [25]. The general impression is that interactions may exist, even though they have been difficult to identify. Attempts to identify gene-gene interactions in T1D in previous studies, e.g. in the recent T1D genome wide association studies [9], [19], [26] have not been fruitful, however, stratifying for known T1D loci while searching for dependent effects at other known or unknown loci may not be the best method. Studies using simulated data have shown that the power to detect risk variants can be increased when allowing for epistasis in addition to single marker effects in e.g. genome-wide association studies [27]. Novel methods taking multiple loci at a time into account may offer possibilities of detecting interactions not detectable by classical methods. Evaluation of suggested interactions is necessary to support novel methods, and by no doubt replication of genetic interactions is important, even though it is currently not obvious how this should be done.

In the current study we have integrated several approaches and our findings support such methods as valuable in searching for yet unidentified genetic and functional interactions involved in the pathogenetic processes of T1D. Evaluation of functionality is by this approach taken into account much earlier than in classical analyses where evaluation of functional significance is typically not performed before the end of a study. The exact consequence of the up- and down-regulations of the proteins in the interaction networks, permanently or transiently, and in relation to T1D, remains to be resolved. Our approach of measuring the enrichment in the interaction modules of T1D associated SNPs is a novel way of seeking also genetic support for several interacting genes eventually combined in biological pathways.

## Materials and Methods

### Ethics statement

Human pancreatic islets were obtained as samples from a multicenter European Union-supported program on beta-cell transplantation in diabetes directed by Professor D. Pipeleers. The program has been approved by central and local ethical committees. Studies including human lymphocytes were approved by the local ethics committee of Copenhagen (KA 94020gm).

Human islet preparations were obtained from nine donors (aged 8–57 years), six were male donors, three were from female donors. Each preparation was stimulated with a mixture of cytokines (TNF-α (5000 U/ml), IFN-γ (750 U/ml) and IL-1β (75 U/ml)) for 48 hours. Lymphocyte RNA was obtained from nine controls (all males, aged 15–35 years and without diabetes) and eight newly diagnosed T1D patients (all males, aged 15–30 years and with duration of T1D<20 weeks from first insulin injection and with continued insulin treatment since). cDNA from human pancreatic islets with and without cytokine stimulation and cDNA from human lymphocytes from controls as well as newly diagnosed T1D patients was used for comparing expression levels. cDNA was prepared from total RNA by oligo-dT-primed reverse transcription, as described by the manufacturer (TaqMan RT reagents, Applied Biosystems, Foster City, CA, USA). Relative expression levels of selected genes were evaluated by use of TaqMan assays. The Low Density Array system (Applied Biosystems) containing assays for the individual genes as well as housekeeping genes was used on TaqMan 7900HT (Applied Biosystems). For evaluation, expression levels of genes were normalized against the average of three human housekeeping genes, *GAPDH, 18S-RNA* and *PPIA*, and evaluated using the delta-delta Ct method [28]. Relative expression levels of genes were for un-stimulated vs. cytokine stimulated islet preparations compared by use of paired t-tests. Expression levels between control and T1D lymphocyte cDNA were compared by f- and t-test. P-values<0.05 were considered statistically significant.

SNPs were mapped to genes/proteins by identifying all SNPs categorized as tagging each gene in the Wellcome Trust Case Control Consortium (WTCCC) genome wide association scan data [9]. We included SNPs 5 kb upstream and 1 kb downstream of each gene, since these regions have been shown to be strongly enriched for gene regulatory elements important for the function of the particular genes [29]. For each gene only the SNP with the lowest p-value was used, to avoid introducing a bias towards genes with many low p-value SNPs in linkage disequilibrium with each other. For each module the significance of the enrichment of SNPs in the best 0.1, 1 and 5 percentile was compared to 1,000 randomly generated protein interaction networks with a similar number of proteins. The random networks were composed of proteins of similar size as the proteins in the actual network tested to normalize against the fact that large genes will have a higher chance of containing T1D associated SNPs in the best percentiles of a study due to their size alone. P-values were adjusted for multiple testing using Bonferroni correction by multiplying the nominal p-values with 12, which is the total amount of tests used in this study. The significance of overlap between genes identified in our analysis and in a paper by D'Hertog et al. [17] was calculated using hypergeometric statistics.
